# Annotations

**Published:** 1898-11-05

**Authors:** 


### Annotations.

### A Plea for a Quinquennial Census.

The Medical Officer of Health for the city of Liverpool has printed, by the request of the Health Committee of that city, a paper in which he calls in question the accuracy of the estimates of the population of Liverpool which are annually published. The population of Liverpool was 629,443 in 1891, and the Registrar-General estimates it at 633,645 for 1898. That is, he assumes that the population of Liverpool has increased at the rate of 600 per annum, or 4,200 in the seven years. The method by which this assumption is reached is simple enough. A certain increase of population having been noted between the years 1881 and 1891, that increase is divided by ten, and a tenth of it is added to every year that has passed since the last census. It is assumed, in short, that when the population of a place has been increasing or decreasing at a given rate during a given census period, that rate will continue during the succeeding period. Now, in many cases the first assumption is erroneous. There may, for special reasons, be a rush of people to a certain place during a certain time, which may not by any means indicate a steady increase in population. This might be best illustrated from America, where sometimes, during the time of a "land boom," thousands will rush to a site, build a city, often on a very elaborate scale, and for a few years give the place the appearance of a busy and thriving town; but from the fact that there is no natural industry to feed the energies of the town, or, in the case of a mining town, the exhaustion of these natural industries, the place may fall as suddenly as it arose, and be left, perhaps, completely deserted. Such extreme cases do not often occur on this side of the Atlantic; but even here sudden increases and decreases in the population of a town are by no means unknown. Estimates of population, based on this assumption, are however, very misleading. What does this matter, it may be asked? If census figures were a mere matter of curiosity it would not matter much. But the efficacy of any sanitary legislation, any philanthropic efforts, are measured by the effect on population, and one's belief in them may be affected by such inaccuracy. To take an example from Liverpool, the Medical Officer of Health says: "Vauxhall Ward formerly was a notoriously unwholesome district. Pent-up alleys and courts, dwellings of the most grossly insanitary construction and condition, were the rule, but for some years past great efforts have been made to ameliorate the conditions of the district, large numbers of insanitary houses have been demolished, streets have been opened up, cottages and artisans' dwellings of the most approved style have been erected, and yet, year by year, thanks to the gross errors in the estimates of population by the Registrar-General's method, the rate of mortality appears to be going up." The discouraging effect of such errors on philanthropic effort can easily be guessed. An annual census of all England is perhaps too great a boon to ask; but in an era when changes are so rapid as ours, is it not possible to get one every five years? This would keep a certain watch on the fluctuations of population, and would thereby act as a guide, more effectual than the present decennial method of reckoning the population, to the movements of industry and the results of any efforts for ameliorating the condition of the people.

### The Slaughter-house Question.

The proposal to abolish the numerous private slaughter-houses which now exist within the metropolitan area, and to substitute for them a small number of public abattoirs, so distributed and arranged as to ensure that the whole of the meat killed in London shall be subjected to efficient inspection, receives much support from the results of certain inquiries which have been made by the medical officer of health of the county of London. The first thing that he shows is that the provisions contained in the Building Act of 1844 would have gone far to terminate the existence of private slaughter-houses in London, but that these provisions were repealed in consequence of the recommendations of a Select Committee of the House of Commons on Noxious Businesses in 1873. It becomes important, then, to ascertain the reasons which led this Committee to make such recommendations, and we cannot but feel that a distinct point is scored in favour of the proposal when it is shown that, owing to the changes which have come over the meat trade during the past quarter of a century, many of these reasons have lost all applicability to the present condition of affairs. Notwithstanding the growth of the metropolis since that time, the number of animals received at the Metropolitan Cattle Market has diminished, while the Foreign Cattle Market at Deptford has been increasingly used. Moreover, there has been a vast development of the trade in dead meat, and along with that of the system of cold storage, so that, as a matter of fact, the number of private slaughter-houses has already very largely diminished, viz., from about 1,500 in 1873 to 467 in 1897, showing that, however useful they may be to their owners, their continued existence cannot be defended on the ground of their meeting a public need. In fact, the percentage of the total meat supply of London which is furnished by the private slaughter-houses is very small indeed. It is possible that, if the proposals which have been made with the object of securing the proper inspection of meat be carried out, the price of London-killed meat may for a time be slightly raised, but we are by no means certain even that this will be the case. The retail price of meat bears such a very distant relation to the amount received by the farmers that it may well be doubted whether an increased expense in slaughtering, which seems to work out to something like half a farthing per pound, will make any difference at all to the retail price. Practically, we fancy the price of English-killed meat is to a large extent regulated by the price and the quality of that which can be imported, and as there is but little doubt that the quality of the latter will improve, we do not anticipate that the institution of inspection will cause any rise in the price of London-killed meat—except perhaps such a rise as may be legitimately due to an increased demand on the part of the public for an article that has been officially inspected and certified to be free from injurious qualities. To this there can be no objection.

